# Comparing immediate and delayed weight bearing in patients with ankle open reduction internal fixation–A protocol for feasibility randomised controlled trial

**DOI:** 10.1016/j.conctc.2024.101304

**Published:** 2024-05-16

**Authors:** Blare Mason, Zohreh Jafarian Tangrood, Jonathan Sharr, Andrew Powell

**Affiliations:** a Division of Orthopaedic Surgery, Christchurch Hospital, Christchurch, New Zealand; b Department of Orthopedic Surgery and Musculoskeletal Research, University of Otago, Christchurch, New Zealand

**Keywords:** Ankle fractures, Weight-bearing, Surgery, Return to work, Rehabilitation

## Abstract

**Introduction:**

Uncertainty regarding the timing of weight bearing following ankle open reduction internal fixation (ORIF) in patients with different ankle fracture patterns remains. Traditional rehabilitation methods, including six weeks of non-weight bearing (NWB), is still a common approach in many hospitals, while some previous evidence has shown immediate weight bearing (IWB) to be beneficial.

**Method:**

32 adult participants with unimalleolar, bimalleolar or trimalleolar ankle fractures and stable fixation following ankle ORIF will be randomly allocated to either Immediate Weight Bearing (IWB) or Delayed Weight Bearing (DWB) groups. Stability of fixation is a subjective assessment made by the operating surgeon at the completion of fixation and is independent of fracture pattern. Participants in the IWB group will be allowed to weight bear as tolerated within 24 h, while participants in the DWB group will remain non-weight bearing for six weeks. Participants’ data including Olerud and Molander Ankle Score, Self-Reported Foot and Ankle Score, SF-36 health survey, time to return to work will be collected. X-rays will be assessed by orthopaedic team members for fixation-related complications including reduction loss, malreduction/malunion, implant failure and non-union. Participants data will be collected at six weeks, three and six-months post-surgery. We will determine the feasibility of a full RCT through assessing the recruitment rate, adherence rate, and drop-out rate.

**Results:**

Not applicable.

This pilot RCT will endeavour to optimise standard rehabilitation protocols post ankle ORIF.

## 1 Introduction

Ankle fractures are one of the most common lower extremity injuries, with an incidence for those requiring hospital admission ranging from 71 to 180 per 100,000 person-years [[1], [2], [3]]. Scott, Jones [4] reported 203,261 cases of ankle fractures admission over 10 years in England with 75% of patients managed surgically with open reduction internal fixation. Despite the high rate of operative intervention, no consensus exists in literature or in clinical practice regarding the optimal post-operative rehabilitation protocol. Post-operative rehabilitation protocols are heterogenous in terms of timing of weight bearing and initiation of ankle motion (immediate, early: 2–3 weeks, and late: 4–12 weeks post-surgery) [5].

Traditional rehabilitation involves ankle non-weight bearing for six to eight weeks before weight bearing as tolerated. This approach aims to allow sufficient time for fracture healing to reduce the risk of fixation failure [6]. Alternative, post-rehabilitation protocols involve early weight bearing with or without early ankle motion within two weeks post-operation, aiming to create mechanical stimulus for bone healing and to improve functional recovery in patients with ankle fractures [6,7]. Despite evidence showing better functional outcomes and shorter recovery time favouring early weight bearing [8,9], concerns still exist regarding potential loss of fracture fixation. This discourages some surgeons from initiating early weight bearing for fear of fracture displacement [10]. Therefore, the traditional non-weight bearing rehabilitation protocols continue to be the most common approach over the past 10 years [5].

Systematic reviews demonstrate studies initiating weight bearing from 24 h to three weeks post-surgery can have better functional outcomes and shorter time to return to work, with no differences in the rate of complications compared with delayed weight bearing [[11], [12], [13], [14]]. However, concerns still exist around the timing of weight bearing in relation to the injury pattern, fixation stability and patients’ medical status [12,15]. Simple transverse or oblique fractures can potentially support weight bearing, where compression improves the chances of bony union compared with spiral or multi-fragmentary fractures [16,17]. Ongoing research in this field may provide further insights into the optimal post-operative rehabilitation protocols appropriate for various ankle fracture patterns.

To the best of our knowledge, despite a range of research aiming to shed light on this issue, no large-scale high-quality study has adequately compared immediate weight bearing following ankle fixation to six weeks non-weight bearing. Smeeing, Houwert [9] conducted an RCT with 115 patients randomised to either group 1 (unprotected immediate weight bearing and immediate ankle motion (within 24 h)); group 2 (protected early weight bearing in a cast (within 10 days) and ankle immobilization); or group 3 (unprotected non-weight bearing and immobilization (for six weeks). The primary outcome of the study showed a higher functional score and shorter time to return to work in patients in the unprotected immediate weight bearing group compared with the protected early weight bearing and unprotected non-weight bearing groups. This study was limited by a relatively small sample size and allowed weight bearing while also allowing range of motion exercises, making the effects of the weight bearing portion of their protocol difficult to isolate.

**Aim:** To conduct a pilot trial recruiting 32 participants with ankle fractures requiring ORIF, to assess the effects of immediate versus delayed weight bearing in the short term. This pilot RCT will test the feasibility of a full RCT through estimating the recruitment rate, adherence rate and drop-out rates for six months following their operation. The follow-on study will be a large-scale and multi-centre RCT to test whether immediate weight bearing following ankle fixation results in improved patient reported outcomes and earlier return to work without an increase in complications compared with patients managed with six weeks of non-weight bearing. The pilot trial will allow us to build the capacity and skills within our institution to effectively lead a multi-centre RCT.

## 2 Methods

### 2.1 Study design

We will conduct a parallel group, superiority designed, and open label pilot RCT, and randomise participants with ankle fracture and open reduction internal fixation to either immediate weight bearing (IWB) or delayed weight bearing (DWB) group post-surgery.

This study will be reported following Standard Protocol Items: Recommendation for interventional trial statement (SPIRIT) [18]. The study will be performed according to the Declaration of Helsinki. This trial has been registered with the Australian New Zealand Clinical Trials Registry (ANZCTR): ACTRN12623001107617. Ethical approval was obtained from Health and Disability Ethics Committees (HDECs). Informed consent will be obtained from all patients, informing them of the study aims, procedures and possible risks.

### 2.2 Study setting

Participants will be recruited between February to December 2024 from Christchurch Hospital, a tertiary trauma centre in Christchurch, New Zealand. We anticipate the full RCT running at this centre, in addition to other secondary and tertiary hospitals in New Zealand.

### 2.3 Eligibility criteria

Patients who are treated with open reduction internal fixation due to a unilateral ankle fractures will be sought for study enrolment. Stable fixation must be achieved by the operating surgeon. While subjective, this is likely to include criteria such as the patient having good quality bone, a good lag screw, and no significant unexpected comminution. Inclusion criteria will be skeletally mature patients (closed distal tibial physes) who are operated on due to closed ankle fractures. There is no upper age limit for inclusion. Fracture patterns will include isolated medial, lateral or posterior malleolus fractures, bimalleolar and trimalleolar fractures. Additionally, ankle fractures treated with an external fixator prior to definitive surgery will be included. These criteria were selected as no study has yet shown the association between the type of fractures and post-surgical outcomes (e.g., Quality of Life, Function scores, complications) [[19], [20], [21]], thus, if the other inclusion criteria are met, patients with different types of fracture will be included. Patients must be physically active (with or without walking aids) in the community before the injury and must speak English or have an interpreter available.

Exclusion criteria will include fractures where stability is still questionable following fixation, as deemed by the operating surgeon. Patients with active infection at the surgical site, any compound fracture, pathological fractures, Pilon fractures, or concurrent injuries that preclude adhering to the follow up protocol will be excluded. We will exclude participants who are unable to follow the protocol due to neurologic disorders (i.e., cognitive impairment, substance abuse, mental illness, intellectual disability, and dense peripheral neuropathy) or significant additional physical impairment (i.e. polytrauma patients with other limb, head or visceral injuries). Any fracture where syndesmosis stabilisation is required will be excluded as post-rehabilitation practices are more conservative with this type of fracture [22]. We will exclude patients whose follow up care will be provided outside of Christchurch Hospital.

### 2.4 Interventions

Patients will receive pre-operative ankle x-rays as their primary imaging modality. CT scans will be obtained if deemed necessary by the orthopaedic team for surgical planning. All patients will be treated by orthopaedic registrars, fellows or consultants according to their preferred techniques. After surgery, all included patients will be placed in a moonboot (also known as a walker boot, or CAM boot). The moonboot consists of a rigid outer shell providing structural support for the foot and ankle, and inner padding to reduce pressure points. They can be removed for monitoring of the wounds. It allows for weight bearing while restricting ankle movement. Patients will be instructed to keep their moonboots on at all times, and to keep their wounds dry. Patients in the IWB group will bear weight as tolerated within 24 h. They will be encouraged to stand or walk without using walking aids. It is envisaged that patients in the IWB group will self-limit the amount of weight they put through their ankle based on their pain tolerance and physical fitness. If necessary, walking aids (crutches, walking frames etc.) will be provided as determined by the treating doctors, nurses, physiotherapists and occupational therapists. Patients in the DWB group will use walking aids (crutches, walking frames etc.) for mobilising and remain non-weight bearing for six weeks prior to allowing weight bearings as tolerated.

At approximately two weeks post-surgery, all patients will have their wounds reviewed in the outpatient clinic. Patients will then have their wounds redressed and be placed back into their moonboot to further 4 weeks.

### 2.5 Data collection

Patient baseline characteristics and hospital data will be collected through electronic medical records during hospitalisation and at discharge by research team members. The data will consist of participants demographics including age, sex, weight, height, BMI, ethnicity, pre-injury level of activity, smoking status, medical comorbidities, and employment status, injury characteristics including date of injury, mechanism of injury, and fracture classification, and treatment details including the date of surgery, type of fixation, and hospital length of stays. We will also record these data from patients who were excluded due to the surgeon deeming fixation unsuitable for immediate weight bearing. This will allow us to further identify if any particular demographic is disproportionately excluded, and minimise the risk of bias in our sample selection for both this pilot, and the full RCT. This will enhance the inclusion and patient selection process in the full RCT.

### 2.6 Outcomes

#### 2.6.1 Primary outcome measures

We will assess recruitment rate, adherence rate, and drop-out rate. We will use an eligibility criteria log to screen participants eligibility. The recruitment rate will be calculated as the proportion of enrolled participants to screened participants and the number of enrolled participants per month. Adherence rate will be identified by the proportion of participants who followed the weight bearing status to all participants at their allocated arm. This will be assessed through participants interviews at the end of the study. Drop-out rate will be measured through the proportion of participants who withdraw from the study after randomisation to those who initially enrolled in the study.

#### 2.6.2 Secondary outcome measures

The Olerud and Molander Ankle Score (OMAS) is a widely used validated patient questionnaire for ankle injuries which scores pain, stiffness, swelling, and various functional abilities from 0 to 100 with 0 representing total impairment, and 100 representing a perfect ankle. The OMAS has high reliability, and high structural validity for assessment of ankle injuries [23]. Minimal Clinical Importance Difference (MCID) is the smallest difference in a patient reported outcome measure that a patient can consider beneficial. The MCID for patients with ankle injuries who underwent ORIF was reported to range between 10.5 and 15.0 points (median 12.5) at three to six months follow-ups [24].

Other secondary outcomes will include a health quality of life survey using SF-36 health survey, and the Self-Reported Foot and Ankle Score (SEFAS). SEFAS is foot and ankle specific Patient Reported Outcome Measure (PROM) scored from 0 to 48, with a higher score indicating a more favourable outcome. It was identified with high content validity and high internal reliability (ICC = 0.93) with no ceiling and floor effect [25]. Time to return to work, time to full weight bearing without walking aids, and return to pre-injury activities will be obtained from patients’ logs in their periodic visits.

Possible post-operative complications will include total and individual complications including venous thromboembolism, wound complications (superficial, and deep wound infection, and wound dehiscence), loss of reduction, fracture malunion/mal-reduction, non-union and implant failure requiring revision surgery. Total complications will be calculated as the ratio of participant with any type of complications to all participants at 6 months post-surgery. Evidence of venous thromboembolism or wound complication will be documented as yes/no during hospitalisation and up to six weeks post-surgery from the patient's medical record. Superficial infection will be as defined by the National Library of Medicine, where infection is confined to the skin and subcutaneous tissues of the incision. Deep wound infection involves infection in the muscles and facial layers [26,27] requiring surgical debridement. Dehiscence of the wound will also be recorded and categorized as a deep wound complication.

At six weeks, three and - six months post-surgery, x-rays will be assessed by the orthopaedic team for fixation-related complications including reduction loss, malreduction/malunion, implant failures and non-union. Reduction loss will be defined as a displacement ≥ 2 mm compared with intraoperative images, or syndesmosis widening. Implant failure will be a breakage of the plate or screws. Non-union will be defined the absence of radiologic or clinical evidence of healing at six months post operation as determined by the orthopaedic team [28,29]. These will be collected from surgeon's x-ray interpretation during patients' clinic visits.

#### 2.6.3 Follow-up time points

**Fig. 1:**
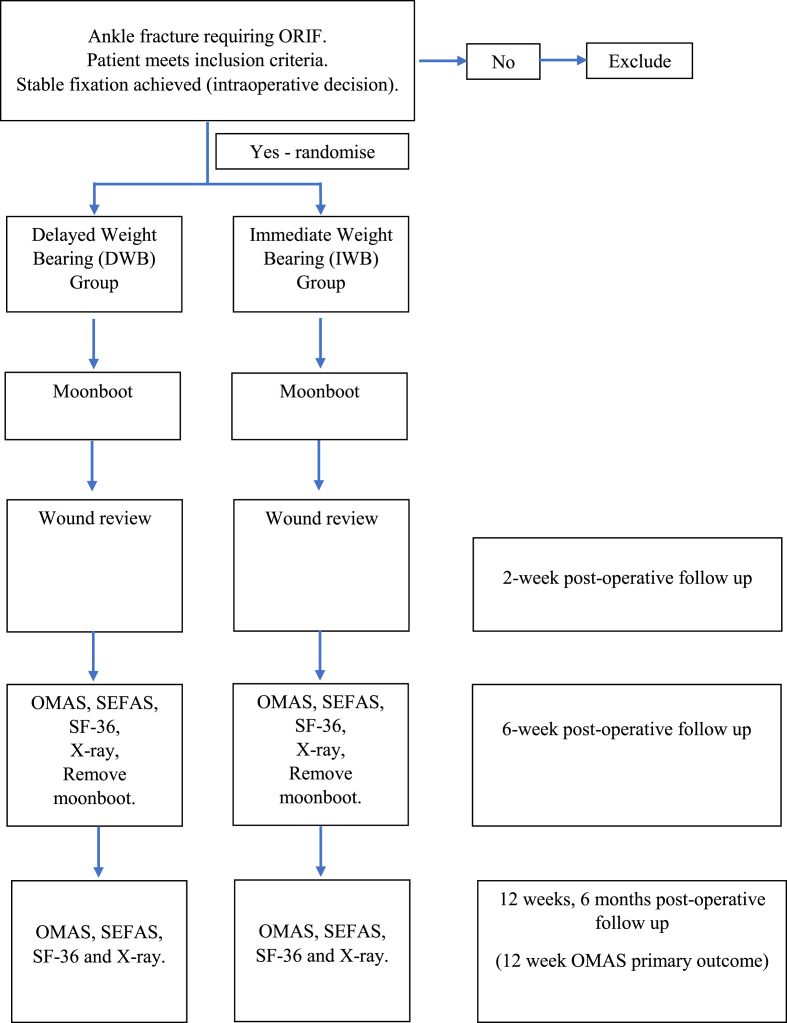
Flow chart showing participants enrolment and follow-ups during the study procedure.

Patient reported outcomes and radiographs will be obtained at six weeks, three and six-months post-surgery. Complications reported by the orthopaedic team will be documented by the research assistants from patient's medical records. The study timeline for participants recruitment and follow-up visits is described in Table 1 and Fig. 1.

**Table 1:** Study timeline.

Assessment	Preoperative	Postoperative	2 weeks	6 weeks	3 months	6 months
**X-ray**	x			x	x	x
**CT-scan**	x					
**Participants screening**	x					
**Informed consent**	x					
**Participants eligibility**		x				
**Allocation**		x				
**Medical history**	x	x				
**Clinic visits (Complication outcomes)**			x	x	x	x
**Research visits (Functional outcomes)**				x	x	x

### 2.7 Recruitment procedure

The research administrator (ZJT) at the centre will make the first approach for screening and explaining the study to potential participants. Eligible participants who are interested in our study will be provided with the information sheet and encouraged to consult with their GPs, family and friends about the study. They will be given adequate time to consider participating in the study prior to their operation. Informed consent for study participation will be obtained prior to surgery. Retrospective data collected from Christchurch Hospital theatre records showed 291 ankle fractures (excluding those with syndesmosis fixation) were operated on during a 12-month period. On the assumption that 50% of these patients would meet the inclusion criteria and agree to participate in our study, we estimate completing recruitment within one year.

### 2.8 Randomisation

A statistician who is not involved in the study will prepare a computer-generated random sequence using blocks of 4 with a 1 to 1 ratio, IWB and DWB groups. At the completion of the operation, and once the stability of fixation is confirmed by the operating surgeon, the research administrator who is independent of the treatment will perform the randomisation using concealed sequentially numbered, opaque and sealed envelopes and will inform the research team of patient allocation group.

### 2.9 Blinding

Due to the nature of the study, surgeons and patients will be unblinded to the group allocation. A research assistant who records the patient clinical outcomes including function and quality of life questionnaires will also be unblinded to the group allocation. Patients' reported questionnaires (objective outcome measures) will not carry the risk of reporting bias, and the blinding status of assessors is unlikely to affect patients’ reporting [30]. Other outcome measures (e.g. time to return to work, time to full weight bearing and post-operative complications) will be collected by unblinded research team members from the patient reporting log, and the wider orthopaedic team, who are involved with patient treatment, but not directly involved with the study. It is possible that those treating team members may carry bias if they are aware of the treatment arm, particularly in the reporting of complications.

### 2.10 Sample size

The minimum clinically important difference for the OMAS was reported 10.5 at three months [24]. Considering the average standard deviation at 16 from prior studies, this gives a sample size of 50 per group for the main trial to achieve 90 % power at a two-sided alpha of 0.05. Given a 20 % drop-out, we will need 120 participants in total for the full trial. In order to minimise the sample size of the pilot and main trial combined, and considering the medium effect size of 0.66, it is recommended that 13 participants per group are recruited for the pilot study [31]. Given a drop-out rate of 20 %, we will need a total of 32 participants for the pilot trial. It is anticipated that these patients will carry over to participate in the ongoing full RCT, pending a successful pilot RCT, ethics approval and appropriate funding.

## 3 Statistical analysis

Descriptive statistics will be used to report participants’ recruitment rate per month, proportion of participants enrolled from the total number screened, adherence rate and the drop-out rate. Specific criteria to determine the feasibility of this study will be a recruitment of two participants per month, the adherence rate of 85 %, and the drop-out rate of ≤20 %.

SPSS (version 28.0; IBM Corporation, Armonk, NY) will be used for statistical analysis. We will report mean and SD for continuous data (OMAS, SEFAS, SF-36, time to full weight bearing, time to return to work and pre-injury activities) and percentage or number for categorical data (total and individual complications).

## 4 Data monitoring

It is possible that obese patients may feel more pain and functional deficit than non-obese patients; however, no associations were identified between increased BMI and post-operative complications (e.g. wound breakdown and loss of fracture fixation) in other studies [32,33]. In this trial, the main investigator (BM) will closely monitor patients with high BMIs, peripheral vascular disease and diabetes in terms of complications and pain presentation. If these complications are identified, patients will be managed according to standard medical treatment practices. Any protocol amendments and decisions about continuation of the pilot trial to the main trial will be discussed and agreed upon by the investigators.

## 5 Expected contribution of this pilot trial to existing knowledge

The pilot trial will help the research team identify possible challenges in study procedure (i.e. recruitment, intervention, data gathering and follow-ups) and fine-tune the study design before commencement of a definitive RCT. Conducting the pilot study will establish the feasibility of a full RCT by facilitating communication and recruitment of other research team members and ensuring successful delivery of the study. Furthermore, it will help us estimate time and cost in completing the definitive RCT. Should the funding become available, we may ask patients in the pilot trial to continue with the research study during the definitive RCT in which they will be followed for 12 months. Finally, it will support the allocation of funding and resources towards a large-scale study.

## 6 Importance of the full RCT

Factors such as low quality of studies and small sample sizes, diversity in post-operative rehabilitation protocols and the lack of adequate knowledge regarding the association between fracture complexity and time to weight bearing have contributed to surgeons’ decisions in identifying a widely accepted post-operation rehabilitation programme. Immediate weight bearing has the potential to benefit patients in earlier return to work and sport, and lower pain and muscle atrophy than those treated with delayed weight bearing [9]. However, a limited number of studies so far have compared immediate weight bearing with delayed weight bearing [9,34,35]. These studies had low sample sizes. Smeeing, Houwert [9], for instance, was unable to reach the estimated sample size and had to terminate the study due to slow recruitment rate and lack of funding. Furthermore, there were discrepancies in reported outcome measures, and findings. Only two studies investigating IWB used similar outcome measures but had conflicting findings, while, Smeeing, Houwert [9] demonstrated significantly better functional outcomes in favour of immediate weight bearing, Honigmann, Goldhahn [34] found no differences between immediate weight bearing and delayed weight bearing. Therefore, the investigation of the efficacy of IWB versus DWB in a study with large sample size and low risk of bias is recommended.

Fracture patterns may also influence the appropriate timing of initiating full weight bearing. It has been recommended that the timing of weight bearing be determined based on fracture pattern and fixation stability [12]. In order to avoid heterogeneity, we will exclude participants with syndesmotic injuries as these participants will need special post-operative consideration. Previous studies have recommended six to eight weeks non-weight bearing for patients with syndesmotic fixation as these patients have demonstrated a higher incidence of post-operative adverse events such as screw breakage or loosening [36]. Excluding these participants may slow down the recruitment rate in our studies; however, will enhance the internal validity of our study. From the full RCT, we will aim to conduct subgroup analysis with respect to different fracture patterns. Fracture complexity will be categorized with respect to ankle fracture pattern, for example simple unimalleolar fractures can be compared with more complex bimalleolar and trimalleolar fractures. Our full RCT will contribute to this body of knowledge by identifying particular fracture patterns which are or aren't amenable to immediate weight bearing.

## Ethics and disclosures

The proposed study was reviewed and approved by the New Zealand Northern B Health and Disability Ethics Committee Ref 2023 EXP 18498 and also received a cultural consultation approval letter from Dr George Haremate, Maori research advisor at Canterbury District Health Board (CDHB) research office. This study will be conducted according to Good Clinical Practice guidelines. The main investigator (BM) who is also an orthopaedic trainee surgeon will not be involved in gaining consent from potential participants. Participants will be reimbursed for their fuel and parking costs during follow-up visits and will receive a gift card at the end of the study. We aim to publish the findings of this study in a peer reviewed journal relevant to this specialty. We will seek to present the findings of the full RCT in national or international orthopaedic meetings.

## Funding

This study has been funded by the Wishbone Orthopaedic Research Foundation of New Zealand, and the 10.13039/501100001505Health Research Council of New Zealand.

## CRediT authorship contribution statement

**Blare Mason:** Conceptualization, Funding acquisition, Investigation, Methodology, Project administration, Writing – original draft, Writing – review & editing. **Zohreh Jafarian Tangrood:** Conceptualization, Formal analysis, Funding acquisition, Investigation, Methodology, Project administration, Writing – original draft, Writing – review & editing. **Jonathan Sharr:** Conceptualization, Investigation, Supervision, Validation, Writing – review & editing. **Andrew Powell:** Conceptualization, Methodology, Supervision, Writing – review & editing.

## Declaration of competing interest

The authors declare no financial and personal competing interests.

## Data Availability

Data will be made available on request.
